# Adverse event profile differences between maribavir and valganciclovir: findings from the FDA adverse event reporting system

**DOI:** 10.3389/fphar.2025.1518258

**Published:** 2025-05-26

**Authors:** Haolin Teng, Shengnan Zhang, Jinyu Yu, Faping Li

**Affiliations:** Department of Urology, The First Hospital of Jilin University, Changchun, China

**Keywords:** maribavir, valganciclovir, cytomegalovirus, adverse event, FAERS

## Abstract

**Background:**

Maribavir and valganciclovir are pharmacotherapeutic options utilized in the management of cytomegalovirus (CMV) infection post-transplantation. Despite their established utility, a comprehensive assessment of their safety profiles in real-world settings remains lacking, particularly with regards to long-term safety outcomes within a sizable cohort.

**Objective:**

The study aims to analyze the adverse event (AE) profiles of maribavir and valganciclovir using data from the U.S. Food and Drug Administration Adverse Event Reporting System (FAERS). This endeavor seeks to juxtapose their respective association strengths and furnish clinicians with pertinent clinical reference points.

**Methods:**

Employing a methodological framework involving the filtration of the FAERS database by specific drugs (maribavir and valganciclovir), AEs attributed to each agent were meticulously cataloged. We applied various disproportionation analysis techniques, including the reporting odds ratio (ROR), proportional reporting ratio (PRR), Bayesian confidence propagation neural network (BCPNN) and multi-item gamma Poisson shrinker (MGPS) algorithms, to identify and quantify potential signals of AEs associated with maribavir and valganciclovir.

**Results:**

There were 999 and 3,454 reports for maribavir and valganciclovir, respectively. Maribavir was primarily associated with the following AEs: dysgeusia (133, 4.07%), taste disorder (127, 3.89%), death (120, 3.67%), fatigue (105, 3.21%), and diarrhea (69, 2.11%). In contrast, the most notable AEs linked to valganciclovir included death (260, 2.59%) and neutropenia (246, 2.59%), leukopenia (175, 1.74%), diarrhea (117, 1.17%), and thrombocytopenia (113, 1.13%). Remarkably, death emerged as an unexpected AE signature for both agents. Key associations were elucidated, notably taste disorder (ROR: 65.23) for maribavir and CMV colitis (ROR: 152.26) for valganciclovir, accentuating distinct AE propensities. Additionally, median onset times for AE manifestation were delineated, with maribavir exhibiting a median onset time of 40 days, compared to 28 days for valganciclovir-associated AEs.

**Conclusion:**

This comprehensive analysis of FAERS data enhances our understanding of the safety. These findings hold implications for ongoing clinical surveillance efforts and provide a foundational basis for subsequent investigations into the safety profiles of these agents.

## Introduction

Cytomegalovirus (CMV) is a pivotal pathogen in the realm of solid organ transplantation and hematopoietic stem cell transplantation (Razonable and Humar, 2019), constituting a significant post-transplantation complication with profound clinical implications (Haidar et al., 2020). The prevalence ranges from 23% to 37% in renal transplant recipients and from 23.5% to 87% in stem cell transplant recipients (Li et al., 2024; Sagedal et al., 2004; De Keyzer et al., 2011). CMV infection significantly increases the risk of transplant failure and mortality (Teira et al., 2016). It also worsens susceptibility to secondary bacterial and fungal infections (Sahin et al., 2016), which further increase the burden of transplantation-related expenses. These challenges are further compounded by the ongoing issue of refractory and drug-resistant CMV infections.

Maribavir was endorsed by the Food and Drug Administration (FDA) in November 2021 and approved by the European Commission in November 2022. It is a significant addition to the therapeutic options for CMV infections. This orally bioavailable benzimidazole riboside is rapidly absorbed and highly bioavailable, rendering it particularly effective in tackling refractory, drug-resistant CMV infections in adult and pediatric populations aged 12 years and older, who have undergone prior treatment with ganciclovir, valganciclovir, and cidofovir post-transplantation (Razonable, 2023). In a Phase II randomized, double-blind study, the most frequently AE was dysgeusia, affecting 65% of patients, followed by nausea (34.2%), and vomiting (29.2%). Dysgeusia resulted in treatment discontinuation in one patient. The study also reported a mortality rate of 27% (32 patients), including four deaths attributed to CMV infections, while the causes of the remaining deaths remain unclear (Papanicolaou et al., 2019). Similarly, a Phase 3 randomized clinical trial identified dysgeusia as the most common AE in the maribavir group, affecting 87 patients (37.2%). Nausea was reported in 50 patients (21.4%), and diarrhea was observed in 44 patients (18.8%) (Avery et al., 2022). In Lauren Ogawa’s study, 7 patients (25.9%) experienced dysgeusia, while 6 patients (22.2%) died from all causes (Ogawa et al., 2024). Additional studies consistently found dysgeusia to be the most common AE in maribavir-treated patients, with 62 patients (43.7%) reporting it, followed by nausea in 24 patients (16.9%) and diarrhea in 23 patients (16.2%) (Blumberg et al., 2024). According to the prescribing information for maribavir, the most prevalent adverse events (AEs) include taste disturbances, nausea, diarrhea, vomiting, and fatigue.

In contrast, valganciclovir has been an FDA-approved agent since June 2001 and has become a cornerstone in treatment of CMV-related diseases (Paya et al., 2004). This evolution is highlighted by its pronounced efficacy in both prophylaxis and treatment settings. The study identified leukopenia as the most common AE of valganciclovir, affecting 19 patients (33.3%) (Abu-Omar et al., 2025). In a separate study of 635 patients treated with valganciclovir, leukopenia occurred in 166 patients (26.1%), while neutropenia was reported in 48 patients (7.6%), with both events being the primary adverse outcomes observed (Rissling et al., 2018). In the current randomized clinical trial, the most frequently reported AEs were leukopenia (37.0%, n = 110), diarrhea (28.6%, n = 85), tremor (17.5%, n = 52), and neutropenia (16.5%, n = 49). A single fatality was recorded, representing 0.3% of the study population. These findings highlight the significant prevalence of these AEs within the trial cohort (Limaye et al., 2023). Chief among the documented AEs linked to valganciclovir, according to its drug label, are neutropenia, anemia, gastrointestinal manifestations (diarrhea, nausea, vomiting), fever, headache, and insomnia.

In this multicenter, double-blind, randomized Phase 3 trial comparing the AEs of maribavir and valganciclovir, the most frequent AEs in the maribavir group were nausea (27.5%, n = 75), anemia (23.1%, n = 63), and vomiting (20.9%, n = 57). In the valganciclovir group, the most commonly reported AEs were neutropenia (52.9%, n = 145), nausea (23.4%, n = 64), and thrombocytopenia (23.0%, n = 63) (Papanicolaou et al., 2024). Although clinical trial data affirm the therapeutic efficacy and safety profiles of maribavir and valganciclovir in managing CMV infections, the limited scope of clinical reports does not fully capture the broad spectrum of AEs, this highlights the need for a thorough exploration of AE signals associated with these agents. This study aims to analyze real-world AE reporting practices for maribavir and valganciclovir within the FAERS. The findings will offer insights to optimize the safe clinical use of these drugs.

## Materials and methods

### FAERS database

Since its inception in 1968, the database has been a cornerstone in post-market surveillance, offering a robust mechanism for monitoring the safety profiles of all FDA-approved drugs and therapeutic biological products. It is a vital tool for the identifying new safety concerns that may have been missed during pre-market clinical trials. Submissions to the FDA come from diverse stakeholders, including healthcare practitioners, manufacturers, and consumers. This diversity ensures a multifaceted and comprehensive data repository (Shu et al., 2022a; Zhao et al., 2024). While the bulk of the reports originate from the United States, the database accommodates contributions from nations worldwide, promoting a globally inclusive approach to pharmacovigilance. Central to the functionality of the FAERS database is its meticulous categorization of AEs and medication errors, which are coded using the Medical Dictionary for Regulatory Activities (MedDRA) terminology. In the context of this investigation, MedDRA version 24.0 was used as the primary lexicon for AE classification. The data file includes seven types of datasets: patient demographic and administrative information (DEMO), drug information (DRUG), coded for AEs (REAC), patient outcomes (OUTC), report sources (RPSR), therapy start dates and end dates for reported drugs (THER), indications for drug administration (INDI), and deleted cases (Yin et al., 2022).

MedDRA’s structured taxonomy, spanning five hierarchical levels from system organ class (SOC) to lowest-level term (LLT), forms the basis for cataloging AEs (Tian et al., 2022; Zhao et al., 2023). The delineation of AEs was categorized in a granular manner at both the SOC and preferred term (PT) levels of the MedDRA hierarchy, thereby providing a more nuanced understanding of AE spectrum (Omar et al., 2021). Notably, FAERS classifies implicated drugs into four distinct categories: primary suspect (PS), secondary suspect (SS), concomitant (C), and interaction (I). The severity of patient outcomes, a pivotal metric in pharmacovigilance, is assessed using critical endpoints encompassing mortality (DE), life-threatening events (LT), hospitalization (HO), disability (DS), congenital anomalies (CA), and other significant medical events (OT) (Steinhart et al., 1994).

### Study procedure

**FIGURE 1:**
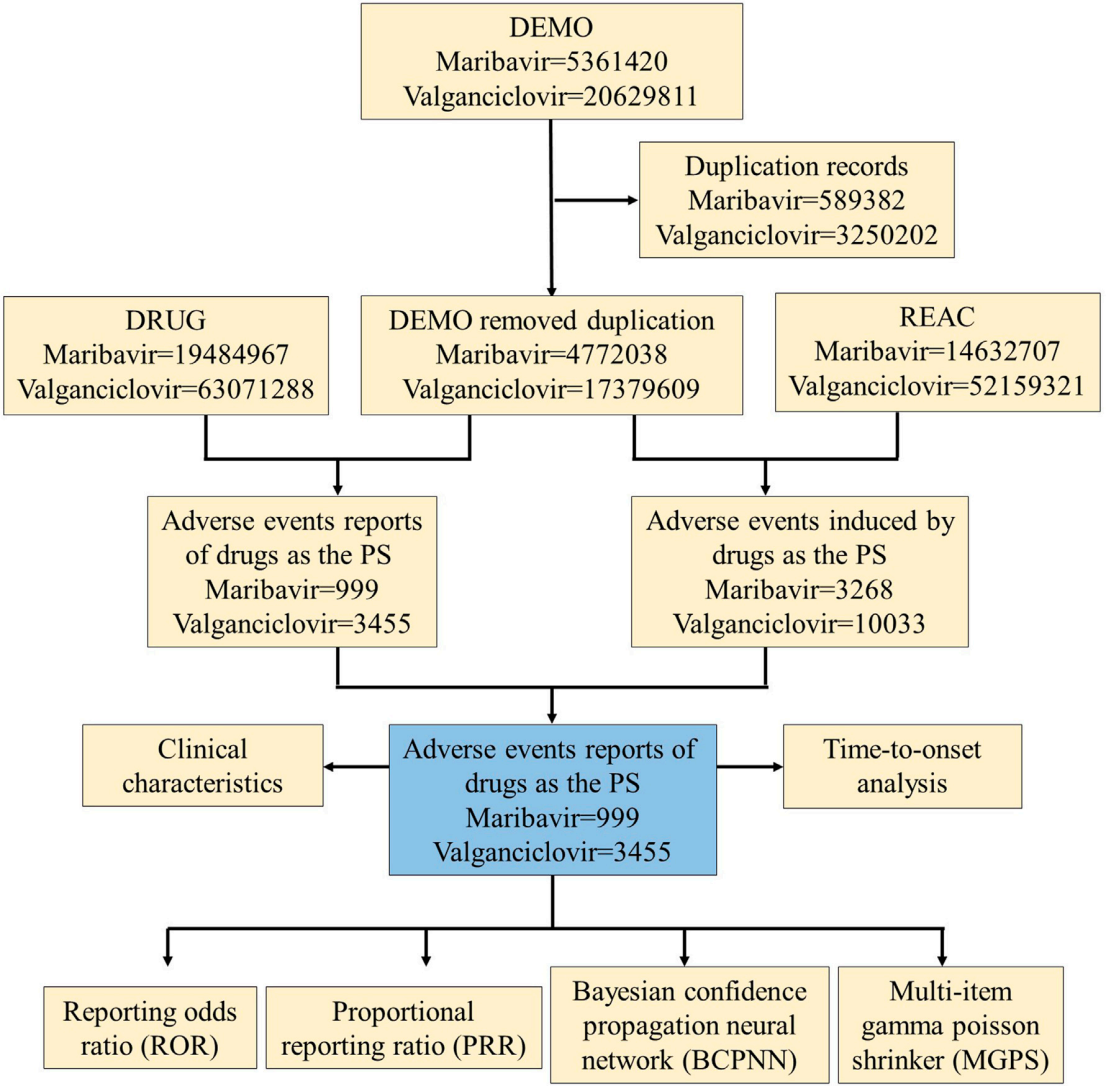
Process flowchart selecting maribavir and valganciclovir-related AEs from the FAERS database.

To assess the incidence and severity of AEs associated with Maribavir and Valganciclovir, we conducted a retrospective pharmacovigilance study. Data were extracted from the FAERS Public Dashboard, an online platform designed for querying the FAERS database. AE reports were identified using keywords corresponding to both generic and trade names of Maribavir and Valganciclovir (“Livtencity” and “Valcyte,” respectively), covering the period from FDA approval to the fourth quarter of 2023. Regarding the reporting sources in the FAERS database, it is important to acknowledge the potential for reporting biases, including underreporting, overreporting, and selective reporting. To mitigate these biases, we considered the diversity of the sources contributing to the reports. We retained all available information, including submissions from consumers, healthcare professionals, physicians, pharmacists, and reports with partial or missing data. This comprehensive approach aimed to minimize the impact of bias and ensure a more robust analysis of the AE data. Given the diverse sources of FDA submissions, it was necessary to address potential duplicate reports. Duplicates were identified and removed by matching the FDA_DT and CASEID identifiers. Additionally, the “role_code” was refined to “PS” to enhance the accuracy of the dataset. AEs were systematically classified according to the MedDRA classification system. The Standardized Organ Class (SOC) and Preferred Term (PT) classifications for AEs related to Maribavir and Valganciclovir were coordinated and served as the primary focus of the data review and research analysis. Clinical characteristics of the AEs associated with these medications were systematically compiled, including demographic factors (sex, age), geographic distribution, reporting sources, time parameters, and clinical outcomes. A detailed flowchart outlining the data extraction, processing, and analysis procedures is presented in Figure 1.

### Time to onset analysis

The delineation of time to onset profiles involved calculating the duration between the “START_DATE” (initiation of maribavir or valganciclovir administration) and the “EVENT_DATE” (manifestation of AEs). Rigorous quality control measures were implemented to exclude reports with data anomalies, erroneous input sequences, or incomplete information.

### Statistical analysis

Statistical analyses were conducted to delineate the association between maribavir and valganciclovir with AEs, leveraging disproportionation analysis as a customary practice in pharmacovigilance studies. Disproportionality analysis is a widely used method for safety signal detection, which is divided into two main categories: Frequentist and Bayesian statistics. Frequentist methods include ROR and PRR, while Bayesian methods include BCPNN and MGPS. They were calculated utilizing established methodologies (Table 1).

**TABLE 1:** Four major algorithms used for signal detection.

Algorithms	Equation	Criteria
ROR	ROR = ad/b/c	lower limit of 95% CI > 1, N ≥ 3
	95%CI = e^ln(ROR)±1.96(1/a+1/b+1/c+1/d)^ ^0.5^	
PRR	PRR = a(c + d)/c/(a+b)	PRR ≥ 2, χ^2^ ≥ 4, N ≥ 3
	χ^2^ = [(ad-bc)^2^](a+b + c + d)/[(a+b)(c + d)(a+c)(b + d)]	
BCPNN	IC = log_2_a(a+b + c + d)(a+c)(a+b)	IC025 > 0
	95%CI= E(IC) ± 2V(IC)^0.5^	
MGPS	EBGM = a(a+b + c + d)/(a+c)/(a+b)	EBGM05 > 2
	95%CI = e^ln(EBGM)±1.96(1/a+1/b+1/c+1/d)^ ^0.5^	

Notes: Equation: a, number of reports containing both the target drug and target adverse drug reaction; b, number of reports containing other adverse drug reaction of the target drug; c, number of reports containing the target adverse drug reaction of other drugs; d, number of reports containing other drugs and other adverse drug reactions.

Abbreviations: 95% CI, 95% confidence interval; N, the number of reports; χ2, chi-squared; IC, information component; IC025, the lower limit of 95% CI of the IC; E(IC), the IC, expectations; V(IC), the variance of IC; EBGM, empirical Bayesian geometric mean; EBGM05, the lower limit of 95% CI of EBGM.

ROR is a disproportionality measure that uses logistic regression to estimate the likelihood of an AE associated with a specific drug relative to all other drugs. It accounts for the total number of reports and adjusts for potential confounding variables. ROR is used to evaluate the strength of the association between a drug and an AE in pharmacovigilance systems. It detects potential safety signals by calculating the ratio of drug occurrences in AE reports to the frequency of the event without the drug (Ren et al., 2025). The PRR is a statistical tool used in pharmacovigilance to assess the relative frequency of adverse drug reactions (ADRs) for a specific drug. By comparing the occurrence of a particular ADR to others, it helps identify potential safety signals and assess the strength of the drug–ADR association (Evans et al., 2001). BCPNN is a Bayesian-based neural network model that predicts drug–ADR associations by leveraging prior and conditional probabilities. It effectively handles small samples or rare events and is robust to noise in ADR data. Compared to traditional methods, BCPNN improves signal sensitivity and accuracy by integrating multiple factors, such as drug, patient, and report timing (Bate, 2007). MGPS focuses on signal detection in large datasets, using shrinkage techniques to adjust effect sizes and minimize false positives. By applying Gamma distributions to centralize estimates (typically around zero), it identifies the most likely signals for further investigation. When combined with BCPNN, the strengths of both methods complement each other, especially in complex drug safety data analysis. This combination reduces random fluctuations and noise in the data, enhancing signal accuracy and improving the overall reliability of safety signal detection (Zhao and Tao, 2024). Each algorithm has unique strengths and limitations. The choice of algorithm depends on the need to balance sensitivity and specificity, depending on the dataset’s characteristics and the importance of minimizing false positives while detecting safety signals. Signals were identified for drugs with at least three AE records. At least one of the four algorithms must indicate a positive signal, with thresholds such as a lower limit of 95% CI > 1, N ≥ 3; PRR ≥ 2, χ2 ≥ 4, N ≥ 3; IC025 > 0 or EBGM05 > 2) (Shu et al., 2022b). Statistical computations, including data preprocessing and deduplication, were conducted using R software (version 4.3.1), ensuring methodological rigor.

## Results

### General characteristics

During the period spanning November 2021 to December 2023, the FAERS database accumulated a total of 5,361,420 AE reports. Following meticulous deduplication procedures, 999 unique AE reports related to maribavir as the PS were identified. Similarly, from 2004 to 2023, a total of 20,629,811 valganciclovir-related AE reports were cataloged in FAERS. Following rigorous data purification, a cohort of 3,455 distinct AE reports linked to valganciclovir as the PS was identified (Figure 1).

Table 2 furnishes an overview of the clinical attributes characterizing events associated with maribavir and valganciclovir. A comparative analysis of maribavir and valganciclovir demonstrated a similar gender distribution, with nearly half of the reports originating from male patients—479 (47.9%) for maribavir and 1,694 (49.0%) for valganciclovir. Notably, the distribution across weight categories demonstrated a smaller proportion of patients within the 50–100 kg and >100 kg brackets in the valganciclovir cohort relative to the maribavir group. Conversely, the age distribution indicated a lower proportion of maribavir-related reports across all age categories, except for the loss group. The median age for maribavir and valganciclovir-associated reports was 60 and 54 years, respectively, predominantly representing the middle-aged demographic.

**TABLE 2:** Clinical Characteristics of Reports with maribavir and valganciclovir from the FAERS Database.

	Characteristics	Maribavir(n) (%)	Valganciclovir(n) (%)
Gender	Number of events	999	3,454
	Male	479(47.9%)	1,694(49.0%)
	Female	415(41.5%)	1,198(34.7%)
	Missing	105(10.5%)	562(16.3%)
Weight (kg)	<50	28(2.8%)	134(3.9%)
	50–100	188(18.8%)	336(9.7%)
	>100	39(3.9%)	18(0.5%)
	Missing	744(74.5%)	2,966(85.9%)
Age(years)	Median (IQR)	60(47–67)	54(34–65)
	<18	14(1.4%)	222(6.4%)
	18–64.9	124(12.4%)	1,036(30.0%)
	65–85	71(7.1%)	419(12.1%)
	Missing	790(79.1%)	1770(51.2%)
Reporter’s type of occupation	Consumer	411(41.1%)	788(22.8%)
	Health profession	329(32.9%)	849(24.6%)
	Physician	223(22.3%)	1,495(43.3%)
	Pharmacist	35(3.5%)	305(8.8%)
	Missing	1(0.1%)	17(0.5%)
Patient outcome	DE	134 (10.0%)	538 (13.1%)
	DS	2 (0.1%)	28 (0.7%)
	HO	421 (31.5%)	1,005 (24.5%)
	LT	5 (0.4%)	128 (3.1%)
	OT	470 (35.2%)	1718 (42.0%)
	CA	-	9 (0.2%)
	RI	-	2 (0.0%)
	Missing	305 (22.8%)	667 (16.3%)
Reported countries	United States	899(90.0%)	1792(51.9%)
	France	40(4.0%)	306(9.4%)
	Canada	11(1.1%)	130(3.8%)
	Spain	8(0.8%)	174(5.0%)
	Great Britain	7(0.7%)	133(3.9%)
	Russia	6(0.6%)	-
	Netherlands	4(0.4%)	-
	Switzerland	3(0.3%)	-
	Germany	1(0.1%)	99(2.9%)
	Australia	-	41(1.2%)
	Belgium	-	40(1.2%)
	Italy	-	93(2.7%)
	Japan	-	183(5.3%)
	Portuga	-	63(1.8%)
	Country Not Specified	20(2.0%)	400(11.6%)

Abbreviations: DE, death; DS, disability; HO, hospitalization-initial or prolonged; LT, life-threatening; OT, other serious; CA, congenital anomaly; RI, required intervention to prevent permanent.

Furthermore, a discernible trend emerged regarding the reporting source of AEs, with maribavir-related events predominantly self-reported by patients, while valganciclovir-related AEs were predominantly reported by healthcare professionals. Regarding serious AE outcomes, mortality and hospitalization constituted the predominant metrics, accounting for (134, 10.0%) and (421, 31.5%) of maribavir-related events, and (538, 13.1%) and (1,005, 24.5%) of valganciclovir-related events, respectively. Notably, valganciclovir was associated with a higher incidence of other serious AE, reporting 1,718 cases (42.0%), compared to 470 cases (35.2%) for maribavir, as observed in the database reports. Geographically, the overwhelming majority of maribavir-related data (899, 90%) emanated from the United States, with a similar trend observed for valganciclovir-related reports.

### Signal detection

We calculated the ROR (95% Two-Sided CI) using the ROR method, and the PRR (95% Two-Sided CI) and χ^2^ tests using the PRR method. This analysis identified a significant association between drug exposure and AEs, with higher ROR values possibly indicating a stronger link to AEs. The combination of PRR and ROR enhances the sensitivity, accuracy, and reliability of safety signal detection. To further validate the signal strength, we applied the Bayesian methods BCPNN and MGPS to calculate the IC(IC250) and the EBGM (EBGM05), respectively. These Bayesian approaches confirm the robustness of the signal detected. The combined use of BCPNN and MGPS addresses the limitations of MGPS when working with small sample sizes and complex data, while MGPS improves the efficiency of BCPNN when handling large datasets.

The analysis ultimately identified 654 and 1,394 signals related to marbavir and valganciclovir-induced AEs, respectively, as detailed in Supplementary Tables S1, S2. The prevalence of the 20 most common PTs associated with each agent is presented in Tables 3, 4, respectively. Predominant AEs observed with maribavir encompassed dysgeusia (133, 4.07%), taste disorder (127, 3.89%), death (120, 3.67%), fatigue (105, 3.21%), diarrhea (69, 2.11%), and nausea (63, 1.93%). In contrast, common AEs linked to valganciclovir included death (260, 2.59%), neutropenia (246, 2.59%), leukopenia (175, 1.74%), diarrhea (117, 1.17%), thrombocytopenia (113, 1.13%), and pancytopenia (100, 1.00%). Notably, these findings are consistent with established prescribing guidelines and drug warnings.

**TABLE 3:** Signal strength of top 20 AEs of maribavir at the preferred terms level in FAERS database.

SOC	PT	n	ROR (95% two-sided CI)	PRR (95% two-sided CI)	χ^2^	IC(IC250)	EBGM (EBGM05)
Nervous system Disorders	Dysgeusia	133	40.59(34.1–48.32)	38.98(38.81–39.15)	4884.44	5.27(3.61)	38.65(33.41)
	Taste disorder	127	65.23(54.56–77.99)	62.74(62.57–62.91)	7613.8	5.95(4.28)	61.88(53.29)
General disorders and administration site conditions	Death	120	2.79(2.33–3.35)	2.73(2.55–2.9)	132.76	1.45(-0.22)	2.72(2.34)
	Fatigue	105	2.48(2.04–3.01)	2.43(2.24–2.62)	89.59	1.28(-0.39)	2.43(2.07)
	Feeling abnormal	26	2.38(1.62–3.5)	2.37(1.99–2.75)	20.65	1.24(-0.42)	2.37(1.72)
	Pyrexia	24	1.38(0.92–2.07)	1.38(0.98–1.78)	2.52	0.46(-1.2)	1.38(0.99)
	Asthenia	21	1.2(0.78–1.84)	1.19(0.77–1.62)	0.67	0.26(-1.41)	1.19(0.83)
	Malaise	20	1.06(0.68–1.64)	1.06(0.62–1.49)	0.06	0.08(-1.59)	1.49(1.06)
Infections and infestations	COVID-19	33	1.11(0.79–1.57)	1.11(0.77–1.45)	0.37	0.15(-1.52)	1.11(0.83)
	Pneumonia	28	1.85(1.28–2.69)	1.84(1.48–2.21)	10.88	0.88(-0.78)	1.84(1.35)
	Sepsis	20	4.07(2.62–6.31)	4.05(3.61–4.49)	45.93	2.02(0.35)	4.05(2.8)
	Urinary tract infection	17	1.85(1.15–2.98)	1.84(1.37–2.32)	6.57	0.88(-0.78)	1.84(1.24)
Investigations	White blood cell count decreased	26	4.02(2.74–5.92)	4.0(3.62–4.38)	58.57	2.0(0.33)	4.0(2.89)
	Haemoglobin decreased	21	4.53(2.95–6.96)	4.51(4.08–4.94)	57.37	2.17(0.5)	4.51(3.15)
	Weight decreased	19	1.28(0.82–2.01)	1.28(0.83–1.73)	1.17	0.36(-1.31)	1.28(0.88)
Gastrointestinal disorders	Diarrhoea	69	2.06(1.62–2.61)	2.03(1.8–2.27)	36.65	1.02(-0.64)	2.03(1.67)
	Nausea	63	1.77(1.38–2.27)	1.75(1.51–2.0)	20.66	0.81(-0.86)	1.75(1.42)
	Vomiting	26	1.27(0.87–1.87)	1.27(0.89–1.65)	1.52	0.35(-1.32)	1.27(0.92)
Respiratory, Thoracic and Mediastinal disorders	Dyspnoea	23	0.86(0.57–1.3)	0.86(0.46–1.27)	0.51	−0.21(-1.88)	0.86 (0.61)
Blood and lymphatic system disorders	Anaemia	22	2.6(1.71–3.96)	2.59(2.18–3.01)	21.59	1.37(-0.29)	2.59(1.83)

Abbreviations: ROR, reporting odds ratio; CI, confidence interval; PRR, proportional reporting ratio; χ2, chi-squared; IC, information component; EBGM, empirical Bayesian geometric mean.

**TABLE 4:** Signal strength of top 20 AEs of valganciclovir at the preferred terms level in FAERS database.

SOC	PT	n	ROR (95% two-sided CI)	PRR (95% two-sided CI)	χ^2^	IC(IC250)	EBGM (EBGM05)
General disorders and administration site conditions	Death	260	1.92(1.69–2.17)	1.89(1.77–2.01)	110.69	0.92(-0.75)	1.89(1.71)
	Pyrexia	66	1.15(0.91–1.47)	1.15(0.91–1.39)	1.34	0.21(-1.46)	1.15(0.94)
Blood and lymphatic system disorders	Neutropenia	246	11.72(10.33–13.3)	11.46(11.33–11.58)	2347.83	3.52(1.85)	11.43(10.28)
	Leukopenia	175	22.06(18.99–25.62)	21.69(21.54–21.84)	3441.88	4.43(2.77)	21.6(19.06)
	Thrombocytopenia	113	6.35(5.28–7.65)	6.29(6.11–6.48)	503.3	2.65(0.99)	6.29(5.38)
	Pancytopenia	100	11.4(9.36–13.88)	11.29(11.1–11.49)	936.99	3.49(1.83)	11.27(9.56)
	Anaemia	85	2.69(2.17–2.33)	2.67(2.46–2.88)	89.2	1.42(-0.25)	2.67(2.23)
	Agranulocytosis	67	24.0(18.86–30.53)	23.84(23.61–24.08)	1460.12	4.57(2.9)	23.74 (19.41)
	Febrile neutropenia	61	5.91(4.59–7.6)	5.88(5.63–6.13)	246.92	2.55(0.89)	5.87(4.76)
	Cytopenia	49	30.56(23.06–40.49)	30.41(30.13–30.69)	1385.86	4.92(3.25)	30.24(23.89)
Gastrointestinal disorders	Diarrhoea	117	1.14 (0.95–1.37)	1.14(0.96–1.32)	2.1	0.19(-1.47)	1.14(0.98)
	Nausea	50	0.39(0.29–0.51)	0.39(0.11–0.67)	48.25	−1.36(-3.02)	0.39(0.31)
	Vomting	49	0.65(0.49–0.86)	0.65(0.37–0.93)	9.29	−0.62(-2.29)	0.65(0.51)
Infections and infestations	Pneumonia	86	1.55(1.26–1.92)	1.55(1.34–1.76)	16.88	0.63(-1.03)	1.55(1.3)
	Sepsis	50	2.73(2.07–3.61)	2.72(2.45–3.0)	54.53	1.44(-0.22)	2.72(2.16)
	Infection	49	2.17(1.64–2.87)	2.17(1.89–2.44)	30.79	1.11(-0.55)	2.16(1.71)
	Cytomegalovirus colitis	47	152.26(113.85–203.62)	151.55(151.26–151.84)	6830.15	7.2(5.53)	147.28(115.48)
Renal and urinary disorders	Acute kidney injury	64	2(1.56–2.55)	1.99(1.74–2.23)	31.55	0.99(-0.67)	1.99(1.62)
	Renal impairment	63	4.73(3.69–6.06)	4.71(4.46–4.95)	184.04	2.23(0.57)	4.7(3.82)
Immune system disorders	Transplant rejection	48	37.86(28.48–50.32)	37.68(37.4–37.97)	1701.9	5.23(3.56)	37.42(29.49)

Abbreviations: ROR, reporting odds ratio; CI, confidence interval; PRR, proportional reporting ratio; χ2, chi-squared; IC, information component; EBGM, empirical Bayesian geometric mean.

A notable finding was the unexpectedly significant incidence of death as an AE. Strong AE associations were identified, with taste disorder (ROR: 65.23) for maribavir and CMV colitis (ROR: 152.26) for valganciclovir emerging as the most significant. In the case of overlapping signals, the reporting odds ratio (ROR) for death (120, 3.67%) and diarrhea (69, 2.11%) associated with maribavir stood at 2.79 and 2.06, respectively. Conversely, for valganciclovir, the ROR for death (260, 2.59%) and diarrhea (117, 1.17%) were 1.92 and 1.14, respectively. Disparities were evident in the incidence and correlation of death and diarrhea, with maribavir exhibiting higher rates compared to valganciclovir.

**FIGURE 2:**
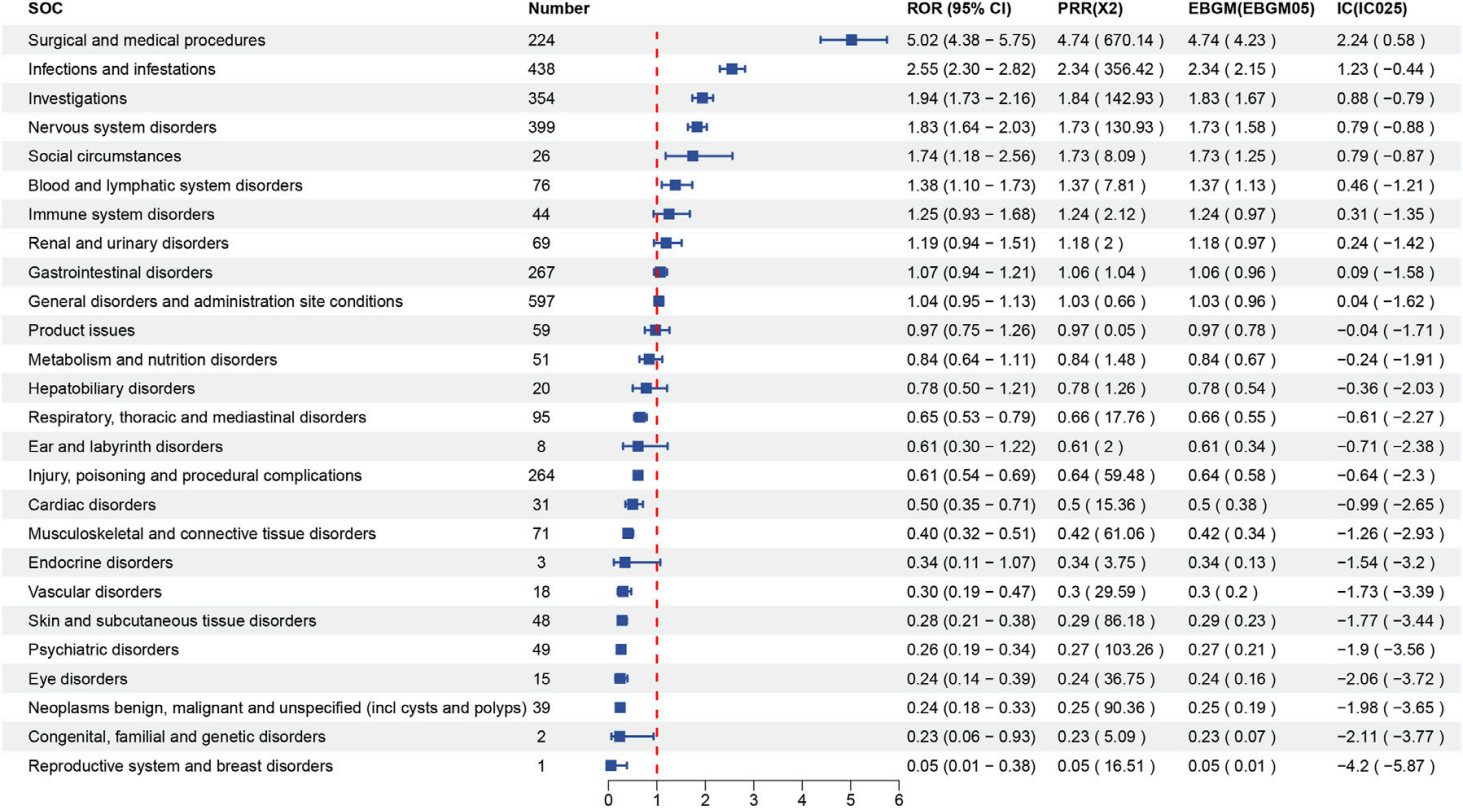
Signal strength of reports of maribavir at the system organ class (SOC) level in FAERS database.

**FIGURE 3:**
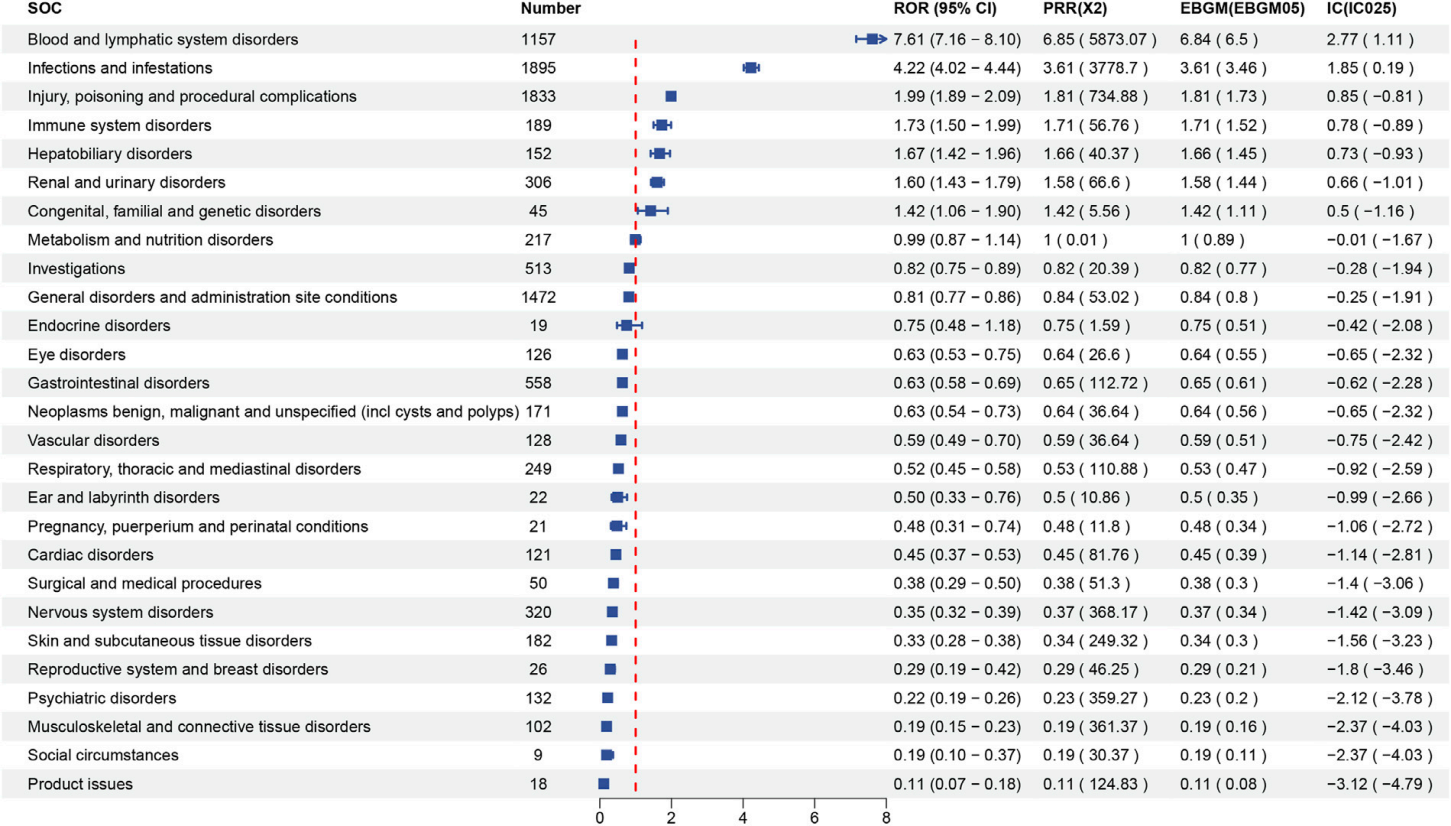
Signal strength of reports of valganciclovir at the system organ class (SOC) level in FAERS database.

Signal strength at the SOC level for maribavir and valganciclovir is shown in Figures 2, 3, respectively. Statistical analysis revealed that maribavir-induced AEs encompassed a spectrum of 26 distinct SOCs. Among these, the top 5 significant SOCs were General disorders and administration site conditions, Infections and infestations, Nervous system disorders, Investigations, and Gastrointestinal disorders. In contrast, valganciclovir-associated AEs manifested across 27 SOCs, with the foremost 5 significant SOCs identified as Infections and infestations, Injury, poisoning and procedural complications, General disorders and administration site conditions, Blood and lymphatic system disorders, and Gastrointestinal disorders.

### Time to onset

The onset timing data for AEs associated with maribavir and valganciclovir were meticulously extracted from the database, with erroneous, incomplete, and unspecified reports systematically excluded. Among the accrued reports, a total of 77 cases involving maribavir and 422 cases involving valganciclovir documented the onset timing of related AEs, as summarized in Table 5. Analysis of the data revealed that the majority of AE occurrences were concentrated within the initial month following administration of both maribavir (36, 46.8%) and valganciclovir (217, 51.4%), with median onset times of 40 and 28 days. The AEs occurring within the first 3 months accounted for maribavir (61, 79.22%) and valganciclovir (337, 79.86%), respectively.

**TABLE 5:** Comparison of time to induce AEs between Maribavir and Valganciclovir.

Group	Maribavir(n)	Valganciclovir(n)
0–30 days	36	217
31–60 days	9	72
61–90 days	16	48
91–120 days	6	17
121–150 days	6	18
151–180 days	-	8
181–360 days	3	21
>360 days	1	21

## Discussion

Previous studies on maribavir and valganciclovir have predominantly focused on clinical trials, mechanistic analyses, and literature reviews, with limited attention exploration of real-world data. This study presents an extensive statistical analysis leveraging the FAERS database to scrutinize potential associations between maribavir/valganciclovir and AEs, providing insights into the post-marketing safety profiles of these drugs. A detailed overview of the AEs associated with maribavir and valganciclovir is provided herein.

Maribavir exerts its pharmacological effect by competitively inhibiting adenosine triphosphate binding at the UL97 site, thereby preventing UL97 phosphorylation and targeting viral protein kinase pUL97 and its substrate, which in turn hinders CMV DNA replication and encapsulation (Grossi and Peghin, 2024; Sun et al., 2024). Clinical trial data consistently identify dysgeusia, nausea, vomiting, and diarrhea (Sun et al., 2024) as the most frequently reported AEs in maribavir-treated patients. This finding is corroborated by Ivy Song’s research (Song et al., 2023). This concordance with both manual records and FAERS database analyses highlights dysgeusia as a prominent AE associated with maribavir administration.

Valganciclovir, a DNA polymerase inhibitor, is rapidly hydrolyzed to ganciclovir after oral ingestion. Intracellularly, ganciclovir triphosphate competes with deoxyguanosine triphosphate as a substrate for viral DNA polymerase, inhibiting viral DNA synthesis and providing anti-CMV activity (Sugawara et al., 2000). Clinical trial data have identified leukopenia, diarrhea, and tremor as the most common AEs associated with valganciclovir (Limaye et al., 2023). Further reports highlight bone marrow suppression as a key AE. This can culminate in anemia, leukopenia, or thrombocytopenia (Blackwell et al., 2021; Morioka et al., 2020).

A comparative analysis of the AEs documented in drug labels and those recorded in the FAERS database revealed similar trends. However, there was a higher frequency of AEs in drug labels. Our statistical analysis examined not only the real-world incidence of AEs associated with maribavir and valganciclovir but also the signal strength and associations at the SOC and PT levels. Notably, the distribution of major associated diseases at the SOC level largely mirrored the findings in the drug labels. According to drug labels, the most frequently reported serious AE associated with maribavir occurred within the Infections and Infestations SOC. In contrast, valganciclovir exhibited a predilection for serious AEs within the Gastrointestinal disorders, Nervous system disorders, and General disorders and administration site conditions SOCs.

In our PT-level analysis, AEs associated with maribavir primarily comprised gastrointestinal disorders, including taste disturbance, diarrhea, nausea, and vomiting. In contrast, valganciclovir-induced AEs were primarily linked to reductions in various blood cell counts. Real-world reports suggest that hemocytopenia is the leading cause for discontinuing valganciclovir, whereas instances of maribavir discontinuation due to this cause are comparatively fewer (Avery et al., 2022; Papanicolaou et al., 2024). Notably, maribavir has shown superiority over valganciclovir in the treatment of cytomegaloviremia (Maertens et al., 2019).

Contrary to the absence of death as a common AE in the drug labels, our data analysis identified death as one of the top AEs for both drugs maribavir and valganciclovir. Maribavir users showed a higher incidence and stronger correlation of death compared to those using valganciclovir. Moreover, diarrhea was a prevalent AE, with higher rates and stronger correlations observed in maribavir users than those receiving valganciclovir. Therefore, valganciclovir may be a more suitable first-choice option for the treatment and prevention of CMV.

The incidence of AEs within the first month of treatment with maribavir and valganciclovir was 46.8% and 51.4%, respectively. Approximately half of the AEs occurred within the initial month of treatment initiation. Nearly 80% of these AEs manifested within the first 3 months of treatment. The rate of AEs associated with both drugs was strikingly similar. Therefore, it is crucial that during the first month of treatment, both patients and healthcare providers closely monitor and document AE symptoms on a daily basis, paying particular attention to the onset, frequency, and severity of these symptoms. Symptomatic treatment was taken for various AEs. Regular testing, including complete blood counts, liver and kidney function assessments, and coagulation profiles, should be performed weekly to detect any potential damage to the blood system or organs, especially for insidious AEs.

In the subsequent second and third months, monitoring of blood and organ functions can be extended to biweekly intervals, with continued daily symptom tracking. After 3 months, monthly assessments can be conducted to evaluate the severity of AEs. Since the beginning of treatment with maribavir and valganciclovir, if mild AEs are identified, and their association with drug dosage is suspected, dose adjustments or substitution with alternative therapies may be warranted. In cases of severe AEs, the drug should be discontinued immediately, and emergency medical intervention should be initiated. Thus, meticulous follow-up and monitoring of AEs are essential for ensuring patient safety when administering maribavir and valganciclovir.

Our study revealed a notable gap in patient weight and age information, which could introduce bias into the analysis results. Most maribavir users were from the United States (899, 90%), while approximately half of valganciclovir users hailed from the same country (1792, 51.9%). This may affect the external validity and universality of the results. Due to the differences in factors such as race, diet and lifestyle between the United States and other regions, the research results may not be fully applicable to patient groups on a global scale. In addition to these personal factors, there are also other potential confounding factors. The relevant differences between the medical system, drug use habits, geographical environment, socio-economic conditions and other regions in the United States may also have an impact on the results. However, these factors were not fully controlled in this study, which might lead to bias in the results and further increase the uncertainty of the results. Future research should consider conducting similar clinical trials or observational studies in other regions, especially among patient groups with diverse geographical environments and different racial and cultural backgrounds, to verify the universality of the research results. Valganciclovir has a broader distribution across reported countries, making its AE analysis more generalizable and representative, which may enhance its safety implications.

While the FAERS database provides extensive data for research and analysis, inherent inaccuracies in data collection and reporting may introduce bias into the analysis results. Furthermore, the database only calculates signal strength without establishing a clear cause-and-effect relationship with AEs. Nevertheless, leveraging AE analysis results remains crucial in mitigating risks associated with clinical treatments.

In summary, our study systematically assessed the potential risks and timing of AEs associated with maribavir and valganciclovir by scrutinizing AE records in the FAERS database. The identification of common, unexpected and serious AEs highlights the need for vigilant patient monitoring during drug interventions. Our findings serve as a valuable reference for further research and clinical management of maribavir and valganciclovir.

## Data Availability

The original contributions presented in the study are included in the article/Supplementary Material, further inquiries can be directed to the corresponding author.
